# Hierarchical Significance of Environment Impact Factor on the Sand Erosion Performance of Lightweight Alloys

**DOI:** 10.3390/ma17163890

**Published:** 2024-08-06

**Authors:** Yuxin Ren, Zhaolu Zhang, Guangyu He, Yanli Zhang, Zilei Zhang

**Affiliations:** 1 National Key Lab of Aerospace Power System and Plasma Technology, Xi’an Jiaotong University, Xi’an 710049, China; renyuxin@stu.xjtu.edu.cn (Y.R.); hegy_22@163.com (G.H.); 2 State Key Laboratory for Mechanical Behavior of Materials, School of Materials Science and Engineering, Xi’an Jiaotong University, Xi’an 710049, China; 3 Science and Technology on Plasma Dynamics Laboratory, Air Force Engineering University, Xi’an 710038, China; 4 China North Engine Research Institute, Tianjin 300400, China; zhangyl1997@126.com (Y.Z.); zhangzileirush@sina.com (Z.Z.)

**Keywords:** lightweight alloys, orthogonal experiment, failure mechanism, erosion rate

## Abstract

This paper addresses the challenge of ranking the factors that affect the erosion resistance of lightweight alloys, with a specific focus on aluminum alloys. A three-factor, four-level orthogonal experimental design was employed to examine the influence of various sand particle sizes, erosion speeds, and sand concentrations on the abrasion qualities of these alloys. Parameters such as mass loss, depth, residual stresses, and failure mechanisms were assessed to determine erosion performance. Analysis of variance (ANOVA) and regression analysis of the three key factors were performed. Our findings resulted in an erosion rate formula: erosion rate = 0.679 sand particle size +0.067 sand concentration −0.002 erosion velocity +0.285. Our findings indicate that particle size is the most significant factor affecting erosion rate, with sand concentration and erosion velocity being secondary factors. The failure mechanism reveals that larger sand particles tend to produce deeper slides, and higher sand concentrations result in an increased number of slides. A lower concentration leads to the appearance of erosion pits. And the test conditions of high concentration and low velocity lead to more serious brittle fractures of the substrate, often accompanied by the appearance of cracks.

## 1. Introduction

The lightweight-alloy turbine impeller plays a critical role in the power system of military vehicles, such as armored vehicles and tanks [1,2,3,4,5]. When these vehicles operate in environments with high concentrations of sand, such as deserts, the high hardness of sand ingested by the engine leads to significant damage to the shape of the aluminum-alloy impeller. This damage results in reduced service life, ultimately impacting the combat performance and durability of the armored vehicles [6,7,8,9,10,11,12].

The sand erosion performance of lightweight alloys is affected by a variety of environmental factors, and the degree of influence varies from one factor to another [1,3,4,13,14,15]. Current research is mainly focused on the erosion performance under the influence of a single factor, and it is deduced that the erosion wear of the material is impacted by factors related to the angle of incidence, impact speed, sand particle size, separation distance, etc. [16,17,18,19,20,21,22,23,24,25,26,27]. For example, Mohammad et al. investigated the erosion performance of flat plate substrates made of aluminum 6063 alloys under different environmental conditions, including varying erosion velocities, angles, spacing distances, and sand particle sizes. They found that lower erosion velocities are associated with higher erosion rates, while the size of sand particles has minimal impact on erosion [28]. Similarly, S. Das et al. examined the wear rate of an Al-Si alloy with varying concentrations and velocities. They observed a correlation between rising sand concentration and an increasing wear rate [29]. However, there is currently a lack of research examining the hierarchical significance of each factor’s impact on the erosion rate in the presence of multiple factors simultaneously. The failure mechanism under the combined effect of multiple factors is still lacking.

In this paper, the impact of environmental factors on the erosion characteristics of 2A70, specifically exploring the influence of sand concentration (0.5–10 g·m^−3^), sand particle size (10–75 μm), and erosion velocity (60–180 m·s^−1^), was investigated. A three-factor, four-level orthogonal test was performed to assess the impact of these variables on the erosion properties of the aluminum alloy and to uncover the failure mechanisms. These findings improve theoretical insights into how environmental factors affect aluminum-alloy materials’ erosion behaviors, providing a reference for subsequent research on lightweight alloys. This study seeks to fill the current research gap regarding the combined effects of various erosion-related factors and to provide valuable insights for the development and optimization of lightweight alloys used in military applications, thereby enhancing the combat performance and durability of armored vehicles and tanks.

## 2. Experimental

To research the erosion behavior of aluminum alloy subjected to varying conditions, aluminum alloy flat plate substrates with a size of 50 × 20 × 3 mm were selected as the research object to carry out orthogonal tests.

**Figure 1:**
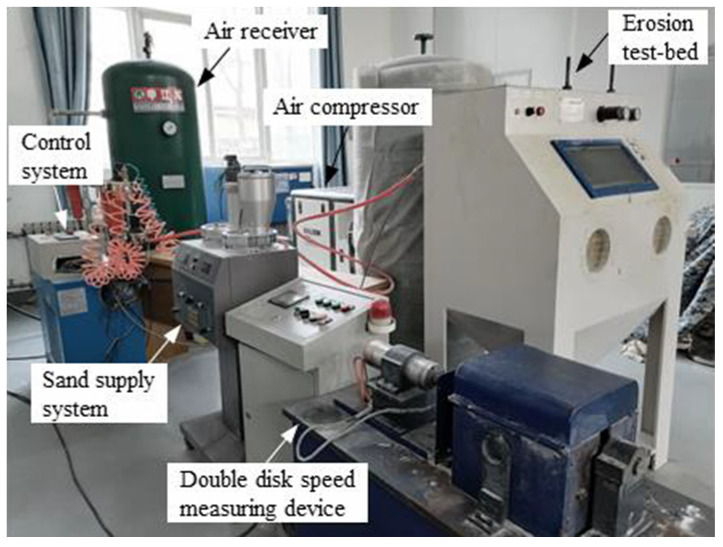
Sand erosion test platform.

The test was executed on the ASTM-G76 Standard-compliant air blast sand erosion test platform [30]. As shown in the Figure 1, the test system mainly consists of the following components: an advanced control system, a stable gas supply system, an accurate sand supply system, a sealed test chamber, and an efficient sand dust recovery device. The control system is operated through an integrated control cabinet, which is responsible for initiating and terminating the sand erosion process, as well as regulating the duration of sand supply. The gas supply system comprises an air compressor and a gas storage tank, with the tank designed to hold a maximum pressure of 6.0 MPa, ensuring the continuity and stability of the gas supply. The sand supply system is equipped with a screw mechanism, and by adjusting its rotation speed, the sand supply can be accurately controlled, thereby regulating the sand concentration in the erosion test. The gas and sand supply pipes converge at the nozzle, and by adjusting the pressure in the gas pipe, the velocity of the sand grains at the nozzle outlet can be precisely controlled. Inside the test chamber, a flat sample holder is installed, which allows for angle adjustment in 15° increments, enabling erosion tests at various angles such as 15°, 30°, 45°, 60°, 75°, and 90° to simulate erosion effects under different conditions. The nozzle has a diameter of approximately 5 mm, and the distance from the nozzle outlet to the sample surface is maintained at about 20 mm, ensuring the accuracy and repeatability of the tests.

**Figure 2:**
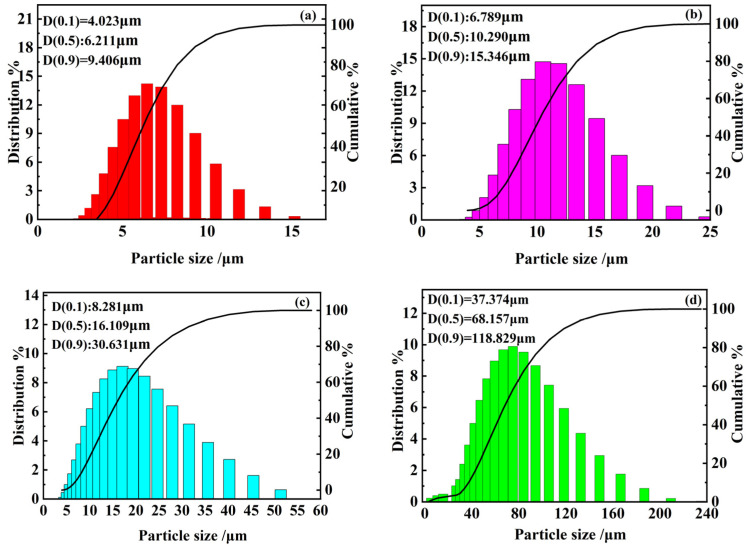
Particle size analysis results for (**a**) 0–10 μm, (**b**) 0–20 μm, (**c**) 0–40 μm, and (**d**) 0–75 μm.

Variables included sand particle size, concentration, and erosion velocity. The erosion test utilized SiO_2_ sand. The angle of erosion was maintained at 90 degrees, while the duration of erosion was varied, being set at intervals of 2 min, 4 min, 6 min, 8 min, and 10 min. Sand composed of SiO_2_ with diverse particle sizes was selected for sand particle analysis employing a laser diffraction-based particle size analyzer. The particle size analysis of the sand is displayed in Figure 2.

**Figure 3:**
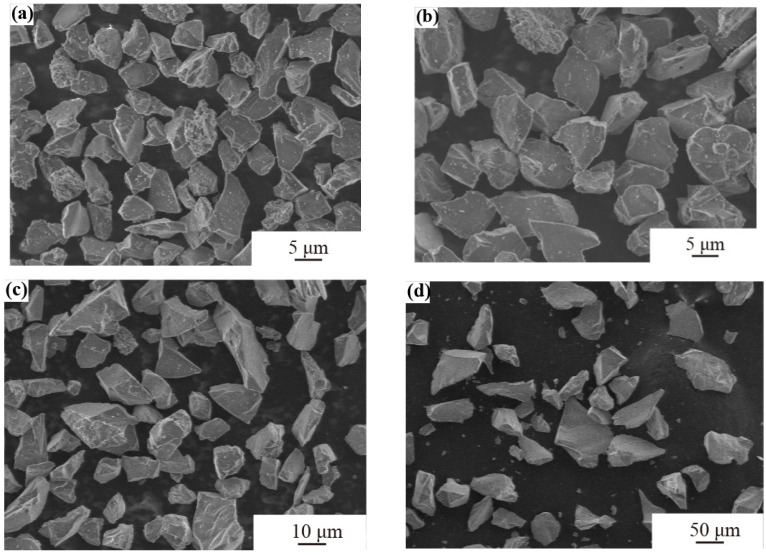
Electron microscopy of sand particle sizes (**a**) 0–10 μm, (**b**) 0–20 μm, (**c**) 0–40 μm, and (**d**) 0–75 μm.

The figure shows that the median particle size values of sand for 0–10 μm, 0–20 μm, 0–40 μm, and 0–75 μm are 6.211 μm, 10.290 μm, 16.109 μm, and 69.001 μm, respectively. Figure 3 presents a detailed examination of the morphological characteristics of dust particles using SEM. It is evident that the particles predominantly exhibit angular shapes with some areas featuring sharp points. The sand particles display a wide size range, demonstrating significant diversity. As the particle size increases, their distribution under SEM tends to become more dispersed. Comparing the four images reveals that when the sand supply is consistent, finer sand particles are more numerous and interact with the sample surface more frequently, leading to a more uniform erosion effect. In contrast, larger sand particles are more sparsely distributed, reducing the frequency of contact with the sample. However, due to the greater mass and momentum of individual particles, their destructive impact on the sample is relatively stronger.

The sand concentration refers to the mass of sand within a given volume. In this paper, four levels of environmental sand concentration were selected, specifically 0.5 g·m^−3^, 2 g·m^−3^, 4 g·m^−3^, and 10 g·m^−3^, in accordance with the actual operating conditions of aluminum alloy within the sand environment of military vehicles. Additionally, four velocity levels, namely 60 m·s^−1^, 90 m·s^−1^, 135 m·s^−1^, and 180 m·s^−1^, were chosen for the test. The variation in erosion velocities was achieved by adjusting the air pressure of the pipeline using the test equipment. The final orthogonal experimental design table is shown in Table 1.

According to the experiment form, the experiment was designed into 16 groups, and each parameter is shown in Table 2.

The surface morphology of sand and the impact damage of an aluminum alloy was analyzed with a field emission scanning electron microscope (FEI Quanta 250, Waltham, MA, USA). Based on Omega Mode (Iso Inclination), the residual stress depth profiles of 2A70 substrate were analyzed (Proto-iXRD, Waterloo, ON, Canda). After chemical stripping with a deionized water solution, the residual stress distribution within the aluminum alloy substrate, influenced by ethanol, butoxyethanol, and perchloric acid, was assessed across its depth. Al (311) crystal plane at 2θ = 149° was selected for residual stress tests in this experiment. Residual stress tests for the erosion marks formed after individual erosion were performed using an X-ray diffractometer. The measurement method employed the tilting fixed ψ method, with the fixed peak method utilizing the half-height and width approach. A voltage of 27 kV and a current of 8.0 mA were used. The depth of erosion damage was quantified through confocal laser microscopy (Smartproof5, Oberkochen, Germany).

## 3. Results and Discussion

**Figure 4:**
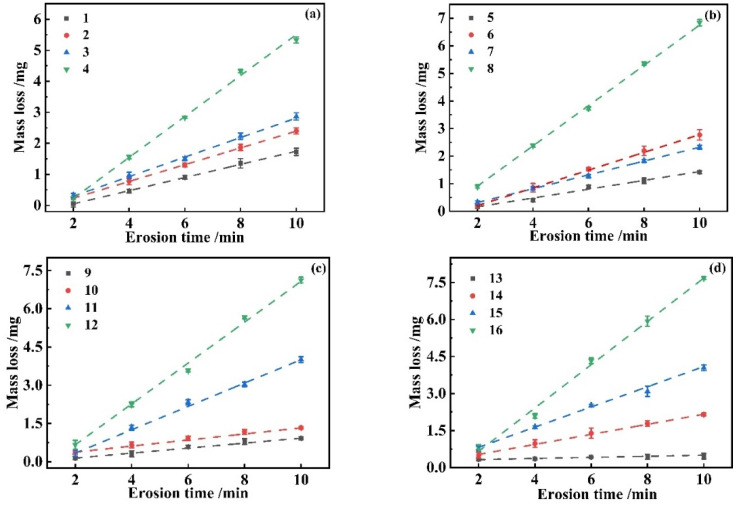
Mass loss of aluminum alloy under different sand particle sizes (**a**) 0–10 μm, (**b**) 0–20 μm, (**c**) 0–40 μm, and (**d**) 0–75 μm.

Figure 4 represents the relationship between sand feed and mass loss under different conditions. The original data mentioned are found in Supplementary Materials, specifically in Tables S1–S4.

The y-axis represents the mass loss, i.e., the reduction within the mass of the substrate after experiencing sand erosion. The slope fitted to the mass loss represents the rate of sand erosion. In Figure 4, the same colors show the same sand concentrations. Grey, red, blue, and green line graphs correspond to 0.5 g·m^−3^, 2 g·m^−3^, 4 g·m^−3^, and 10 g·m^−3^ sand concentrations, respectively. Figure 4a displays the mass loss of the substrate due to erosion by 0–10 μm particles. In Figure 4a, the slope of the green line is significantly larger than the other three lines. In Figure 4b, the mass loss of the substrate under continuous erosion for sand sizes 0–20 μm is shown, and, unlike the other plots, the slopes of test 6 and test 7 are closer to each other. And when the erosion time is shorter, the concentration of sand is larger (4 g·m^−3^), and the erosion velocity is larger (180 m·s^−1^), the slope value is higher. When the sand supply is larger, on the contrary, the large concentration and high velocity of the test conditions instead result in a lower the slope value. This phenomenon suggests that both velocity and concentration have an effect on mass loss. Figure 4c shows the mass loss incurred by the substrate under 0–40 μm sand erosion. Test 9 and Test 10 in Figure 4c have slopes that are also relatively close to each other. Figure 4d shows that the greater the concentration and the lower the velocity, the greater the value of its mass loss. Comprehensive comparison of Figure 4a–d shows that mass loss (d) > (c) > (b) > (a), i.e., sand particle size is correlated with mass loss, which escalates as the sand particle size enlarges throughout the erosion process. Upon comparing the same color and particle size, it is observed that a higher sand concentration correlates with a greater mass loss for the green samples. Conversely, grey samples exhibit a lower mass loss at lower sand concentrations. This indicates that erosion rate increases with sand concentration, illustrating a direct relationship between sand concentration and erosion rate. E. Avcu’s investigation into titanium alloys revealed a direct proportionality between erosion velocity (ranging from 60 m·s^−1^ to 100 m·s^−1^) and mass loss, highlighting the significant impact of velocity on erosion outcomes [31]. Similarly, R. Sahoo’s research on the erosive wear of solid particle titanium alloys identified erosion velocity as the most critical factor affecting the wear rate [32]. Drawing parallels from these studies, it is reasonable to hypothesize that aluminum alloys may exhibit similar behavior, where erosion velocity significantly influences the erosion rate. This aligns with our findings, suggesting a broader applicability of the observed relationship between erosion velocity and erosion rate across different metallic materials.

**Figure 5:**
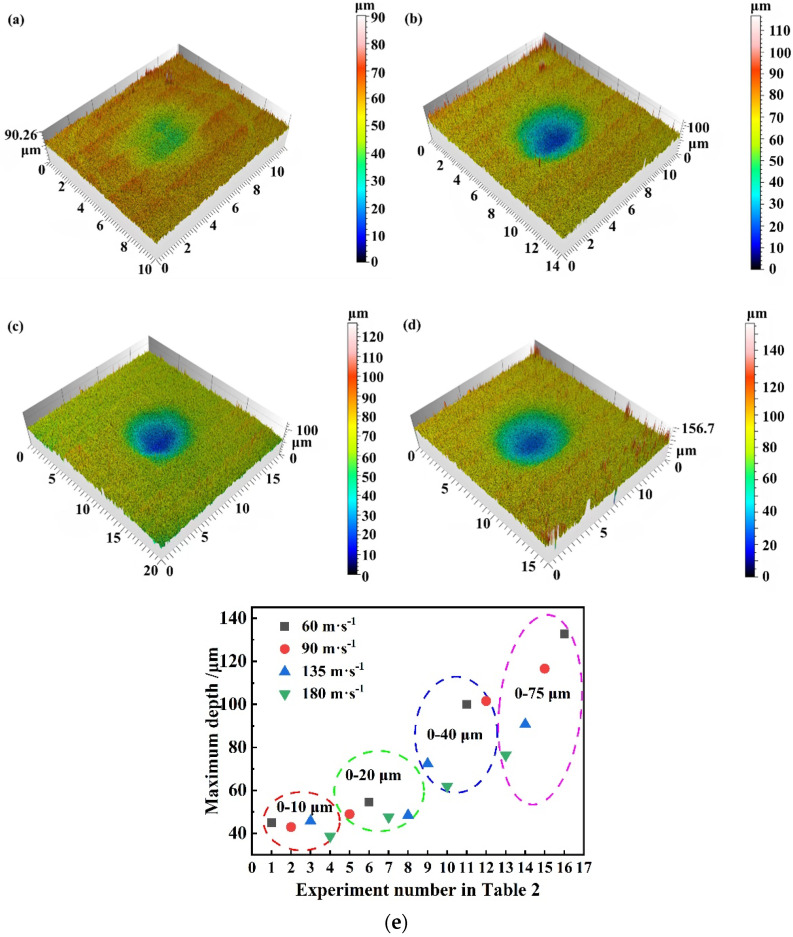
Surface morphology of substrates at different concentrations and velocities for sand particle sizes 0–75 μm at (**a**) 0.5 g·m^−3^ concentration, 180 m·s^−1^ velocity; (**b**) 2 g·m^−3^ concentration, 135 m·s^−1^ velocity; (**c**) 4 g·m^−3^ concentration, 90 m·s^−1^ velocity; (**d**) 10 g·m^−3^ concentration, 60 m·s^−1^ velocity; and (**e**) erosion depth for different experiment numbers.

Figure 5a–d depict the surface morphology of substrates exposed to differing sand erosion conditions, represented by varying sand concentrations and velocities, all using sand particles measuring 0–75 μm.

Figure 5a demonstrates that when the substrate is eroded at a high speed of 180 m·s^−1^ with a relatively low sand concentration of 0.5 g·m^−3^, the depth values are lower, at only 38.61 μm. As the concentration of sand increases and the velocity decreases, the erosion depth becomes greater. Figure 5d indicates that when the erosion velocity is the lowest, and the sand concentration is the highest, the depth of the erosion pits is the greatest. Figure 5e demonstrates the erosion depth under different test conditions. The horizontal coordinates divided the erosion depth into four regions according to the sand size 0–10 μm, 0–20 μm, 0–40 μm, and 0–75 μm, highlighting the correlation between erosion depth and sand particle size. In addition, Figure 5e categorizes the depths of erosion at various velocities, demonstrating the erosion resistance under different erosion velocities of light alloys. As the erosion velocity increases, the erosion depth is instead lower. This phenomenon is more obvious especially when the value of particle size is larger, i.e., the erosion velocity is negatively correlated with the erosion depth. Therefore, the larger the erosion velocity, the lower the erosion depth. At the same time, Figure 5e reveals that the erosion depth and sand particle size show a positive correlation; oval area 4 corresponds to the largest depth value, and oval area 1 corresponds to the smallest depth value. Considering the mass loss, it can be hypothesized that sand particle size and sand concentration have a positive correlation with erosion performance, whereas erosion velocity has an inverse correlation. Stack et al. proposed that velocity affects the mechanism of action of erosion–corrosion and concluded that the greater the velocity, the lesser the erosion rate [29,33]. Similar to the findings in previous papers, the erosion velocity is negatively correlated with the wear rate due to increased mutual collision and rebound, as well as reduced mobility of the eroded particles.

**Figure 6:**
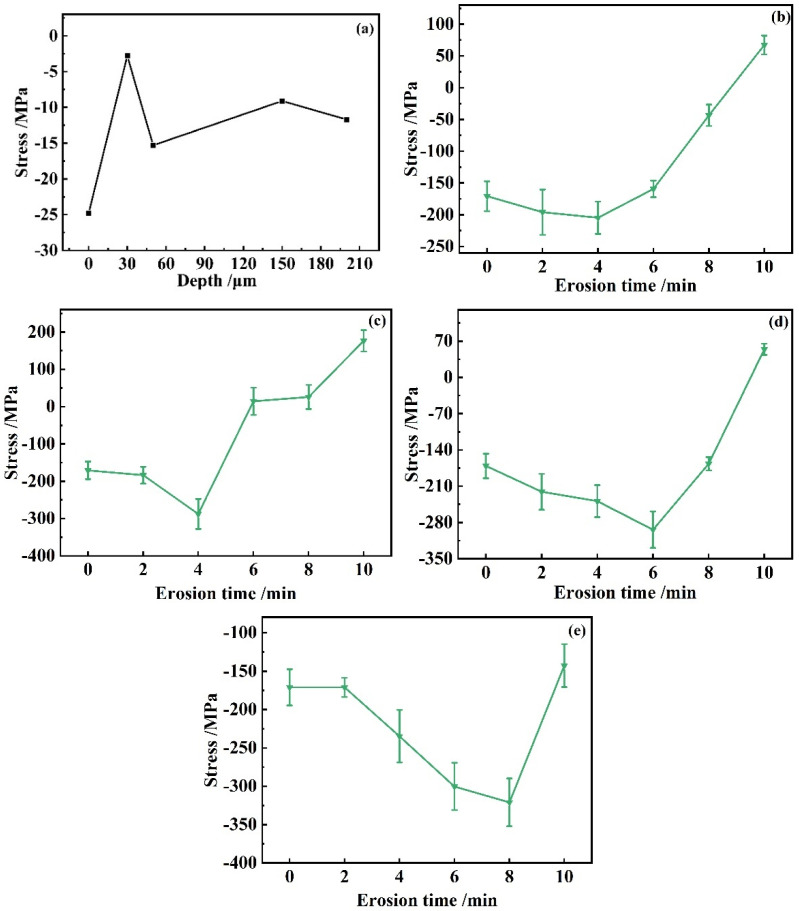
Residual stress values under the same concentration (10 g·m^−3^), different sand particle sizes, and erosion velocities. (**a**) residual stress variation with depth in aluminum alloy substrate; (**b**) 0–10 μm, 180 m·s^−1^; (**c**) 0–20 μm, 135 m·s^−1^; (**d**) 0–40 μm, 90 m·s^−1^; (**e**) 0–75 μm, 60 m·s^−1^.

Figure 6 displays the residual stresses in the substrate and under different test conditions.

Figure 6a illustrates how residual stresses change across different depths in the aluminum alloy substrate. In Figure 6a, residual stresses with increasing depth first show negative values, i.e., residual compressive stresses are generated in the aluminum-alloy material without receiving erosion. With the gradual increase in depth, the residual stress values appear to oscillate around zero. This pattern is consistent with an M-shaped curve and aligns with prior research on the variation of residual stress with depth in aluminum alloys [34]. Figure 6b–e show the results of residual stresses for sand particle sizes of 0–10 μm, 0–20 μm, 0–40 μm, and 0–75 μm, respectively. Figure 6b–d shows that after the aluminum alloy is subjected to erosion, in addition to the residual compressive force generated on the surface of the material due to the introduction of compressive stresses within the aluminum alloy substrate, a phenomenon of residual tensile stresses is subsequently generated within the aluminum alloy substrate. Figure 6e shows that with an increase in the size of the sand particles, the residual stress value is always negative. The explanation is attributed to the enlargement of sand particle sizes, as the substrate undergoes more severe plastic deformation. That is consistent with a shot peening-like effect. The overall trend of the residual stresses for all four groups shows a decrease and then an increase, which also indicates that the increase in the erosion time leads to the failure behavior of crack expansion on the surface of the substrate, which ultimately leads to the change in the residual stress value. Wang studied the influence of shot peening on the erosion–corrosion characteristics of nickel-aluminum bronze. Results indicate that shot peening and erosion–corrosion similarly affect the material, leading to increased hardness and the development of compressive residual stress [35]. In this paper, the residual stress value varies as erosion time increases and exhibits a pattern of initially decreasing followed by an increase. This trend is able to represent the side effect that the surface of the material under erosive conditions gradually fails with the increase in erosion time. Wang’s paper as well as our findings emphasize the complex effects of erosive wear processes on the properties of different materials. This comparison validates our results and complements the trend in hardness under erosion.

Figure 6b–e illustrate an increasing amplitude in the reduction in residual stress values, with the transition point from decrease to increase varying across the datasets. Specifically, Figure 6b records this trend change at an erosion time of 4 min, corresponding to a decrease of 33.9 MPa. Figure 6c shows a similar timing for the trend change, with a more pronounced decrease of 116.8 MPa. As we progress to Figure 6d, the trend change is observed at an erosion time of 6 min, with a decrease of 123 MPa. In Figure 6e, the inflection point is delayed to an erosion time of 8 min, with the maximum decrease recorded at 150.1 MPa. The observed increases in the amplitude of stress reduction and the shifts in the trend reversal point suggest a correlation with the sand dust particle size. Larger particles are likely responsible for more significant plastic deformation on the material surface during the erosion process, leading to an initial increase in residual compressive stress. However, as erosion time extends, the surface begins to exhibit crack propagation, causing the stress state to evolve from compression to tension, which is reflected in the overall trend of the residual stress values.

Table 3 describes the erosion rate outcomes for three distinct factors—sand particle size, sand concentration, and erosion velocity—across four levels, providing a clear indication of each factor’s relative impact on the erosion rate. The analysis of the erosion rate values reveals that the highest erosion rate, at 0.857 mg·min^−1^, occurs under the conditions of a sand particle size of 0–75 μm, sand concentration of 10 g·m^−3^, and erosion speed of 60 m·s^−1^. In contrast, the lowest erosion rate, assessed at 0.016 mg·min^−1^, is found when the sand particle size is 0–75 μm, the sand concentration is 0.5 g·m^−3^, and the erosion velocity is 180 m·s^−1^, indicating superior erosion resistance under these conditions. With regard to the particle size of sand (Factor I), it is observed that as the particle sizes increase from 0–10 μm to 0–20 μm, 0–40 μm, and 0–75 μm, the corresponding erosion rate ranges increase from 0.211–0.642 mg·min^−1^ to 0.157–0.726 mg·min^−1^, 0.097–0.805 mg·min^−1^, and 0.016–0.857 mg·min^−1^, respectively. This trend suggests that the peak erosion rate of sand increases significantly with increasing particle size. In examining sand concentration (Factor II), a similar upward trend is evident. Within groups of experiments sharing identical conditions apart from varying sand concentrations, each incremental increase in concentration correlates with a commensurate uplift in the erosion rate. For example, from tests 1 to 4, 5 to 8, 9 to 12, and 13 to 16, under identical particle size conditions, the erosion rate steadily rises with increasing concentration. As for erosion velocity (Factor III), the data present a different pattern. For example, comparing test groups 1, 4, 9, and 13, with the same concentration, the erosion rate value decreases as the size of sand particles gradually increases, and the erosion velocity rises while the value of the erosion rate decreases. These observations suggest a theory that an inverse relationship exists between the erosion velocity and the erosion rate, a phenomenon that needs more research. From the 16 sets of tests with three factors and four levels, it can be hypothesized that the three factors, namely, different sand particle size, sand concentration, and erosion velocity, affect the erosion performance of aluminum alloys. 

In this paper, IBM SPSS Statistics 27 software is used to analyze the significance of the data to explore the relationships between three different variables. When analyzing using the software, the three factors are analyzed quantitatively. In this case, when calculating the sand particle size in Factor I, the data were analyzed according to the corresponding median particle size D (0.5), i.e., 0–10 μm was calculated according to 6.211 × 10^−6^ m, 0–20 μm was calculated according to 10.290 × 10^−6^ m, 0–40 μm was calculated according to 16.109 × 10^−6^ m, and 0–75 μm was calculated according to 68.157 × 10^−6^ m.

The orthogonal design table was analyzed by ANOVA and the findings can be observed in Table 4 below. In Table 4, the *p*-value obtained in the ANOVA is less than 0.05, indicating that the factors have a significant effect on the variables. That is, the three factors of sand particle, sand concentration, and erosion velocity all exert a considerable impact on the erosion rate of aluminum alloy. At the same time, the regression analysis was conducted on the experimental data, and the analysis results are displayed in Table 5. The |t| > 2 obtained in the regression analysis indicates that the impact of the independent variable on the dependent variable is significant. This indicates that the erosion rate can be significantly influenced by controlling the sand particle size, sand concentration, and erosion velocity to control and predict the behavior of the system. The final regression analysis showed that the unstandardized coefficients were 0.679, 0.067, and −0.002, respectively, while the standardized coefficients are 125.954, 0.067, and −0.0017 respectively. The unstandardized coefficients indicate the relationship between the amount of change in the dependent variable, erosion rate y, and the three independent variables, sand particle size, concentration, and erosion velocity. The final equation of the linkage between the erosion rate and the three factors was derived as shown in Equation (1) below. The standardized coefficients were able to identify the order of influence of each independent variable on the dependent variable. And the absolute values of the three factors are ranked as follows: sand particle size is the largest, sand concentration is the second, and erosion velocity is the smallest. The order of magnitude of the most significant effect on the erosion rate was caused by the sand particle size, the second largest sand concentration, and the smallest erosion velocity.

Finally, the relationship can be simplified as shown in Equation (1):

(1) y=0.679a+0.067b−0.002c+0.285

where *a* represents the median sand particle size, denoted as D(0.5)/m, *b* is the sand concentration/g·m^−3^, which is associated with the sand concentration, *c* is the erosion velocity/m·s^−1^, and *y* is the erosion rate. Equation (1) displayed that the sand particle size and sand concentration show a positive correlation with the results of the erosion rate of aluminum-alloy substrates. The larger the particle size, the larger the mass loss and the greater the erosion rate, indicating a deterioration in erosion performance, which may be analyzed because the larger sand particle size is more likely to cause larger pits and scratches on the surface. The higher the sand concentration, the more significant the mass loss and the higher the value of the erosion rate, and analysis suggests that the reason may be that the greater sand concentration means that the density of sand particles within a specific area is greater. This leads to an increase in the impact area of the surface, which ultimately leads to a greater loss of quality. At the same time, the sand erosion velocity and erosion rate show a negative correlation; the faster the sand erosion velocity, the lesser the erosion rate, and the slower the sand erosion velocity, the greater the erosion rate.

Three sets of validation tests were carried out to verify the above equations’ rationality. The selected test parameters are shown in Table 6 below. Validation test 1 was compared with the original test 1 (sand particle size 0–10 μm, sand concentration 0.5 g·m^−3^, erosion velocity 60 m·s^−1^), and the particle size was changed. Validation test 2 was compared with the original test 16 (sand particle size 0–75 μm, sand concentration 10 g·m^−3^, erosion velocity 60 m·s^−1^), changing the erosion velocity. Validation test 3 changed the sand concentration compared to the original test 13 (sand particle size 0–75 μm, sand concentration 10 g·m^−3^, erosion velocity 90 m·s^−1^).

**Figure 7:**
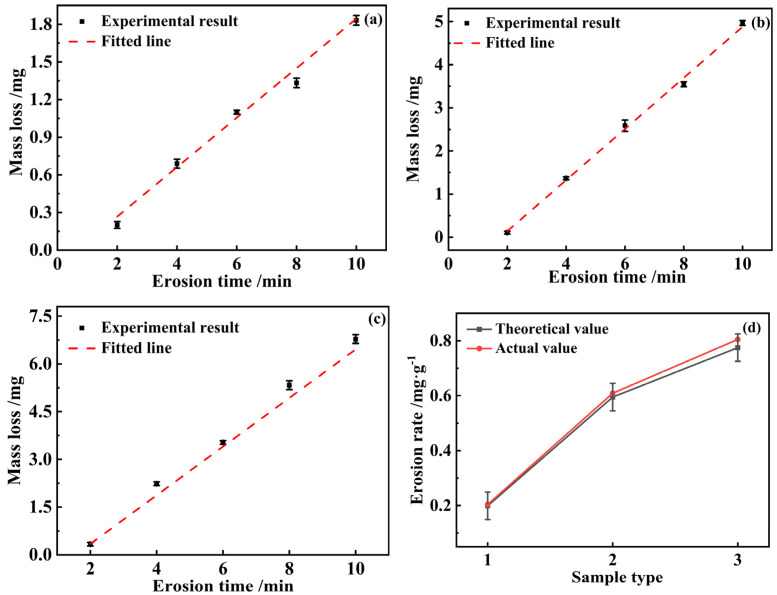
Erosion rate results of the validation test; (**a**) validation test 1, (**b**) validation test 2, (**c**) validation test 3, (**d**) comparison of theoretical and actual results.

A similar experimental approach was taken for the validation tests. The mass loss at each erosion time was measured and subsequently fitted. The fitting results are shown in Figure 7 below.

Figure 7a–c show the mass loss produced by validation tests 1, 2, and 3 when subjected to the same sand particle size, different sand concentrations, and different velocities, respectively. Comparing Figure 7b,c, both sand concentrations are the same and the overall erosion rate value is lower for the higher velocity and higher for the lower velocity. Substituting the test conditions of validation tests 1, 2, and 3 into Equation (1) as shown in Figure 7d, the theoretical erosion rates were finally obtained to be about 0.199 mg·min^−1^, 0.595 mg·min^−1^, and 0.775 mg·min^−1^, and the actual erosion rates were about 0.204 mg·min^−1^, 0.609 mg·min^−1^, and 0.805 mg·min^−1^, respectively, with the fluctuation in error rates not more than 5%. As shown in Figure 7d, they are all within the error bars, so the formula is reasonable.

**Figure 8:**
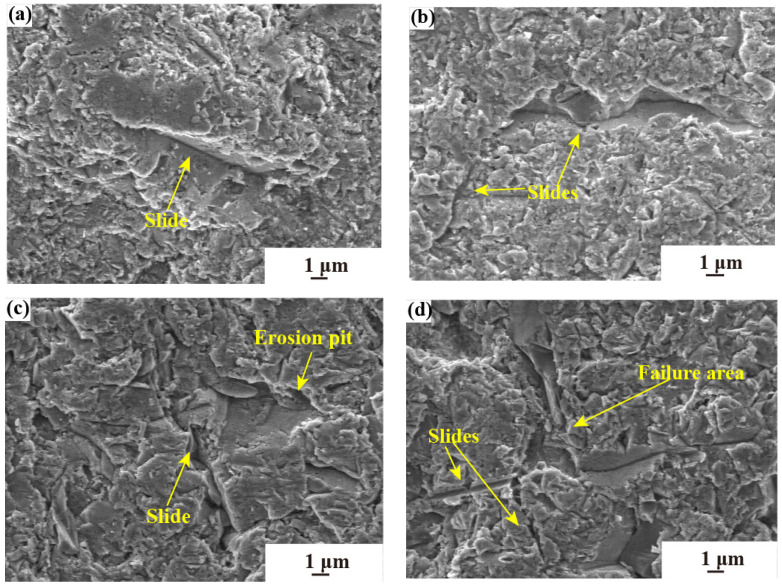
The erosion characteristics of different sand particle sizes; (**a**) 0–10 μm size, 0.5 g·m^−3^ concentration, 60 m·s^−1^ velocity; (**b**) 0–20 μm size, 0.5 g·m^−3^ concentration, 90 m·s^−1^ velocity; (**c**) 0–40 μm size, 0.5 g·m^−3^ concentration, 135 m·s^−1^ velocity; (**d**) 0–75 μm size, 0.5 g·m^−3^ concentration, 180 m·s^−1^ velocity.

Figure 8 illustrates the surface morphology of aluminum-alloy substrates subjected to different sand particle sizes under consistent erosion duration, aiming to delve into the failure mechanisms of the substrates.

Figure 8a–d indicate that when the sand concentration is 0.5 g·m^−3^, the aluminum-alloy substrates are subjected to the impact of different sand particle sizes of 0–10 μm, 0–20 μm, 0–40 μm, and 0–75 μm, respectively. As illustrated in Figure 8a–d, there are slippage tracks. Due to the irregular shape of sand particles, they may collide with other particles during the erosion process, which will cause the deviation of the incidence angle before contacting the substrate. The deviation can cause sand particles to create slippage tracks on the surface of the lightweight alloy, which can contribute to the formation of wear slides. The depth and width of the erosion slide on the substrate are different. When the size of the sand particles increases, the slide is more obvious, i.e., the depth is deeper. In Figure 8a,b, the existence of small particles at low sand concentrations results in a relatively uniform failure pattern. There are only one or two slides. In addition, the slide lengths shown in Figure 8a,b are short, consistent with the low particle size of sand, and no large damage area is formed. Figure 8c shows that with large particle size, low sand concentration, and high-velocity conditions two forms of failure, pits and slides, occur simultaneously. Compared with the previous two figures, the failure at this time is more serious. Figure 8d shows that there are two large guide rails forming a cross and forming a failure area. That is, the failure is more serious at this time, and the erosion rate is the highest under this test condition. The failure shown in Figure 8 is consistent with the formula in this paper. It is argued that there exists a positive correlation between sand particle size and erosion rate; specifically, as the sand particle size increases, the substrate mass loss and the erosion rate both escalate. At the same time, in the process of sand erosion, the sand particle size plays a dominant role. 

**Figure 9:**
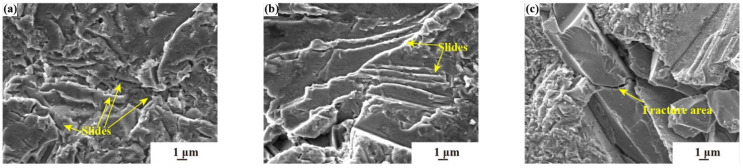
The erosion characteristics of different sand concentrations; (**a**) 0–40 μm size, 2 g·m^−3^ concentration, 180 m·s^−1^ velocity; (**b**) 0–40 μm size, 4 g·m^−3^ concentration, 60 m·s^−1^ velocity; (**c**) 0–40 μm size, 10 g·m^−3^, 90 m·s^−1^ velocity.

Figure 9a,b demonstrate that when the sand particle is 0–40 μm, as the sand concentration value increases, erosion gradually begins to occur at the same place and at the same time, with the formation of multiple slides, and the higher the sand concentration value, the greater the number of slides.

Figure 9a shows that when high speeds and large concentrations are present, there is the creation of a slipway in addition to the creation of a squeeze lip at the same time. In Figure 9a, the slides start to diverge from each direction, and the reason is still due to the collision of sand particles, which causes a certain deviation in the incidence angle. Figure 9b shows that at lower speeds, there is no squeeze lip generation and the form of failure is only characterized by the simultaneous occurrence of multiple slides. In Figure 9b, the slides diverge in fewer directions than in Figure 9a, but the slide length is longer, and the slides in both directions eventually lead to the formation of a large failure area. Figure 9c shows that the experimental conditions of high concentration and low velocity lead to brittle fracture. The fracture area is shown in the figure, accompanied by the appearance of cracks.

**Figure 10:**
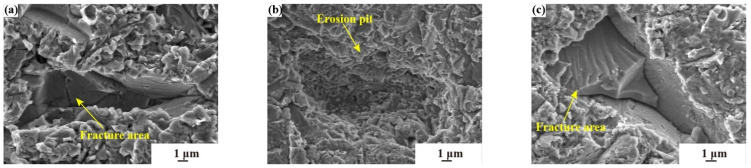
The erosion characteristics of different velocities; (**a**) 0–75 μm size, 4 g·m^−3^ concentration, 90 m·s^−1^ velocity; (**b**) 0–75 μm size, 0.5 g·m^−3^ concentration, 135 m·s^−1^ velocity; (**c**) 0–75 μm size, 10 g·m^−3^, 60 m·s^−1^.

Figure 10a–c show that when the particle size of sand is 0–75 μm, the failure form is mainly manifested in the appearance of large pits and brittle fractures.

The respective experimental groups’ erosion rates are significantly higher than those of the other experimental groups, measured at 0.409 mg·min^−1^, 0.197 mg·min^−1^, and 0.857 mg·min^−1^, respectively. Figure 10a shows that under high concentration, large particle size, and low velocity erosion, brittle fractures occur, accompanied by cracks and erosion pits. Figure 10b illustrates that with a reduction in both concentration and velocity, the material exhibits the formation of considerable pitting, yet without a significant occurrence of fracturing. And Figure 10c reveals that under conditions where the sand concentration is at its peak and the velocity at its minimum, a substantial area of brittle fracturing emerges, leading to critical failure. This aligns with the highest observed erosion rate, which is quantified at 0.857 mg·min^−1^. Such conditions result in a conjunction of brittle fractures and pit formation, confirming the earlier theoretical analysis that indicates this to be the scenario of the most severe deterioration.

## 4. Conclusions

This study focused on lightweight alloys, with aluminum alloy as a representative example, and explored the ranking of the influence of multiple concurrent factors—such as sand particle size, sand concentration, and erosion rate—on the erosion rate of these substrates. By designing a three-factor four-level orthogonal test, the sand particle size was divided into four levels of 0–10 μm, 0–20 μm, 0–40 μm, and 0–75 μm, the sand concentrations were 0.5 g·m^−3^, 2 g·m^−3^, 4 g·m^−3^ and 10 g·m^−3^, respectively, and the erosion velocity of the sand particles was divided into four levels of 60 m·s^−1^, 90 m·s^−1^, 135 m·s^−1^, and 180 m·s^−1^.

1. A series of 16 orthogonal tests were conducted to gather data on mass loss, depth, residual stress, and erosion rate under varying conditions. It was observed that larger sand particles and higher sand concentrations were associated with increased erosion rates, and a positive correlation was found between erosion rate and pit depth. The residual stress of the aluminum alloy matrix exhibited an M-shaped curve, consistent with the shot peening effect. Additionally, the trend in the residual stress values with erosion time are all consistent with a decrease followed by an increase, which is consistent with the failure mechanism of the substrate surface.
2. The experimental data were subjected to theoretical analysis using variance and regression analysis to determine the erosion rate. It was revealed that the influence of sand particle size on erosion rate outweighed that of sand concentration and erosion velocity. The relationship was mathematically formulated as *y* = 0.679*a* + 0.067*b* − 0.002*c* + 0.285, where ‘*y*’ represents the erosion rate, ‘*a*’ is particle size, ‘*b*’ is sand concentration, and ‘*c*’ is erosion velocity. The model’s error rate was less than 5%, substantiating its validity.
3. Furthermore, an analysis of the erosion mechanism highlighted that larger sand particles resulted in deeper erosion slippage and more prominent erosion scars. Under consistent sand particle size, higher sand concentration resulted in multiple slides occurring simultaneously in the same location, leading to a broader range of failures. When the erosion particle size was the same, the lower concentration led to the appearance of erosion pits. And the test conditions of high concentration and low velocity led to more serious brittle fractures of the substrate, often accompanied by the appearance of cracks.

This paper contributes to the field of erosion research by providing valuable insights into the erosion performance, analysis methodology, mathematical modeling, and erosion mechanisms of aluminum-alloy substrates under various conditions. The findings have practical implications in industries where erosion is a concern, such as military vehicles.

## Figures and Tables

**Table 1:** Three-factor design table.

	Factor I (Sand Particle Size/μm)	Factor II (Sand Concentration/g·m^−3^)	Factor III (Erosion Velocity/m·s^−1^)
Level 1	0–10	0.5	60
Level 2	0–20	2	90
Level 3	0–40	4	135
Level 4	0–75	10	180

**Table 2:** Table of experimental design.

Factors Experiment Number	Sand Particle Size/μm	Sand Concentration/g·m^−3^	Erosion Velocity/m·s^−1^
1	0–10	0.5	60
2	0–10	2	90
3	0–10	4	135
4	0–10	10	180
5	0–20	0.5	90
6	0–20	2	60
7	0–20	4	180
8	0–20	10	135
9	0–40	0.5	135
10	0–40	2	180
11	0–40	4	60
12	0–40	10	90
13	0–75	0.5	180
14	0–75	2	135
15	0–75	4	90
16	0–75	10	60

**Table 3:** Erosion rate results.

Factors Experiment Number	Sand Particle Size/μm	Sand Concentration/g·m^−3^	Erosion Velocity/m·s^−1^	Erosion Rate/mg·min^−1^
1	0–10	0.5	60	0.211
2		2	90	0.266
3		4	135	0.317
4		10	180	0.642
5	0–20	0.5	90	0.157
6		2	60	0.316
7		4	180	0.251
8		10	135	0.726
9	0–40	0.5	135	0.097
10		2	180	0.119
11		4	60	0.459
12		10	90	0.805
13	0–75	0.5	180	0.016
14		2	135	0.197
15		4	90	0.409
16		10	60	0.857

**Table 4:** ANOVA results.

Source of Variance	Sum of Squares of Deviations	Degrees of Freedom	Mean Square	F-Value	*p*-Value	Significance
Sand particle size	0.015172	3	0.005057	0.002529	0.00785	*p* < 0.05
Sand concentration	0.01277	3	0.004257	0.002128	0.00834	*p* < 0.05
Erosion velocity	0.016042	3	0.005347	0.002674	0.00766	*p* < 0.05
D Error	0.004596	3	0.001532	0.000766	0.00964	

**Table 5:** Results of regression analysis.

Ratio					
Model	Unstandardized Coefficient		Standardized Coefficient	t	|t|
	B	Standardized Error	Beta		
Independent Variables	0.285	0.029	0.283	2.090	|t| > 2
Sand particle size	0.679	0.000	125.954	3.167	|t| > 2
Sand concentration	0.067	0.010	0.067	3.142	|t| > 2
Erosion velocity	−0.002	0.000	−0.0017	−3.073	|t| > 2

**Table 6:** Parameters of the validation test.

Factors Experimental Number	Sand Particle Size/μm	Sand Concentration/g·m^−3^	Erosion Velocity/m·s^−1^
Validation test I	0–75	0.5	60
Validation test II	0–75	10	180
Validation test III	0–75	10	90

## Data Availability

The original contributions presented in the study are included in the article/Supplementary Materials, further inquiries can be directed to the corresponding author.

## Supplementary Materials

The following supporting information can be downloaded at: https://www.mdpi.com/article/10.3390/ma17163890/s1, Table S1: Original data of mass loss from erosion tests for groups 1–4; Table S2: Original data of mass loss from erosion tests for groups 5–8; Table S3: Original data of mass loss from erosion tests for groups 9–12; Table S4: Original data of mass loss from erosion tests for groups 13–16.
