# Supplementary material for: Hierarchical Significance of Environment Impact Factor on the Sand Erosion Performance of Lightweight Alloys
Source: Materials (Basel). 2024 Aug 6;17(16):3890. doi: 10.3390/ma17163890 (PMC11355729; doi:10.3390/ma17163890)
Supplement: Supplementary file 1 [file materials-17-03890-s001.zip › materials-3106335-supplementary.pdf]

The following tables are presented as supplementary data.

Table S1. Original data of mass loss from erosion tests for groups 1-4

Erosion time /min	1		2		3		4	
	Mass loss	Error value	Mass loss	Error value	Mass loss	Error value	Mass loss	Error value
2	0.06	0.12	0.23	0.06	0.32	0.07	0.23	0.06
4	0.46	0.06	0.78	0.12	0.97	0.1	1.56	0.06
6	0.90	0.06	1.30	0.06	1.5	0.06	2.83	0.03
8	1.36	0.15	1.87	0.1	2.23	0.1	4.33	0.06
10	1.73	0.12	2.4	0.1	2.87	0.12	5.33	0.1

Table S2. Original data of mass loss from erosion tests for groups 5-8

Erosion time /min	5		6		7		8	
	Mass loss	Error value	Mass loss	Error value	Mass loss	Error value	Mass loss	Error value
2	0.17	0.06	0.2	0.06	0.32	0.05	0.9	0.06
4	0.40	0.06	0.85	0.15	0.83	0.05	2.38	0.06
6	0.89	0.05	1.53	0.06	1.27	0.06	3.74	0.06
8	1.11	0.1	2.19	0.17	1.82	0.06	5.36	0.07
10	1.42	0.05	2.77	0.19	2.32	0.07	6.84	0.12

Table S3. Original data of mass loss from erosion tests for groups 9-12

Erosion time /min	9		10		11		12	
	Mass loss	Error value	Mass loss	Error value	Mass loss	Error value	Mass loss	Error value
2	0.13	0.06	0.37	0.06	0.33	0.12	0.67	0.17
4	0.31	0.12	0.66	0.12	1.33	0.1	2.25	0.1
6	0.59	0.06	0.92	0.08	2.33	0.1	3.58	0.06
8	0.79	0.12	1.17	0.09	3.03	0.09	5.67	0.06
10	0.92	0.06	1.33	0.05	4.01	0.12	7.13	0.12

Table S4. Original data of mass loss from erosion tests for groups 13-16

Erosion time /min	13		14		15		16	
	**Mass loss**	**Error value**	**Mass loss**	**Error value**	**Mass loss**	**Error value**	**Mass loss**	**Error value**
2	0.33	0.04	0.5	0.12	0.77	0.1	0.83	0.1
4	0.35	0.03	0.97	0.15	1.63	0.06	2.1	0.1
6	0.42	0.02	1.39	0.21	2.51	0.06	4.33	0.12
8	0.43	0.1	1.78	0.12	3.09	0.21	5.93	0.21
10	0.46	0.12	2.15	0.06	4.04	0.12	7.68	0.06
